# Clinical Surgery of the Hospital of La Pitié

**Published:** 1844-07

**Authors:** 


### BRITISH AND FOREIGN MEDICAL REVIEW,

FOR JULY, 1844.

### PART FIRST.

### Analytical and Critical Reviews.

### Art. I.

*Clinique Chirurgicale de l'Hôpital de la Pitié.* Par J. Lisfranc. Tome troisième.—*Paris,* 1843.

*Clinical Surgery of the Hospital of La Pitié.* By J. Lisfranc.—*Paris,* 1843. Third Vol. 8vo. pp. 748.

The present volume, which completes M. Lisfranc's work, is—with the exception of two chapters, one on tumours adjacent to but not connected with the female organs of generation, the other on hemorrhoids,—entirely devoted to certain diseases of the uterus and of its appendages. We shall not lay before our readers a detailed analysis of the entire volume, as the greater part of its contents necessarily consist of generally known facts, but we shall endeavour to notice whatever seems valuable either from being more or less novel, or from bearing on important or unsettled points of pathology or practice.

*Fibrous tumours of the uterus.* The first chapter treats of fibrous tumours of the uterus, a disease not yet fully elucidated, notwithstanding the labours of Bayle, Bichat, Roux, Cruveilhier, Dupuytren, Lee, and others. The history given of the pathological anatomy of the affection agrees in the main with that given by the writers whom we have just enumerated, and as anything on that head requiring remark may be more conveniently noticed when considering the chapter on polypus of the uterus, we shall only advert to M. Lisfranc's views respecting the symptoms, diagnosis, and treatment of the malady.

The account of the symptoms produced by fibrous tumours, though rather diffuse, is not very accurate. M. Lisfranc does not, for example, mention how materially the symptoms vary according to the situation of the tumour, and it might be readily inferred from his account, that those symptoms are the same, whether the growth projects towards the peritoneum or encroaches on the cavity of the uterus; while it is to the latter form of the disease that the description given is chiefly applicable. On such points, however, we shall not dwell; our object is not to examine the history of the affection in detail, but to state M. Lisfranc's doctrines respecting its symptomatology and treatment, which, though scattered through fifty-two pages, are comprised in the three following propositions: First, that there are no signs by which the existence of a fibrous tumour of the uterus, can in any case be positively ascertained during life. Secondly, that, in consequence of the impossibility of forming an accurate diagnosis, every supposed case of fibrous tumour of the uterus should be treated as if it were a curable induration of that organ. And thirdly, that, though the true fibrous tumour is always incurable, its progress may often be arrested for years, by means of such treatment. M. Lisfranc claims those opinions as his own undoubted discoveries, and alleges, as we shall see, that he has sustained no small persecution for having been bold enough to promulgate them. How far this claim is founded, we shall briefly examine, after having stated the facts and reasoning adduced by our author in support of his views, and this we shall do as nearly as possible in his own words.

The rational symptoms produced by fibrous tumours of the uterus, merely announce the existence of some disease of that organ, without specially indicating any particular malady; but there are three symptoms, to any of which, where present, M. Lisfranc attaches very considerable importance, viz. the occurrence of expulsive pains at variable periods, but more especially a few days before or after menstruation; the existence of menorrhagia, usually very obstinate, and, when controlled, extremely liable to return;—and above all, to this, viz.

"That the volume of womb may diminish under appropriate treatment, and after a time again augment in size. It is not very unusual to see this diminution and subsequent increase of the hypertrophy of the uterus occur several times in succession. The phenomenon, I think, may be thus satisfactorily explained. The medicines administered disperse, but only for a time, either partially or entirely, the hypertrophy of the tissue of the uterus, caused by the presence of the fibrous tumour. These last-mentioned signs are, in my opinion, very important, they are not, it is true, pathognomonic, but they afford very strong presumptions in favour of the existence of a fibrous tumour." (p. 13.)

M. Lisfranc next considers the physical signs, or those ascertainable by the sight and the touch, and maintains that they are equally inconclusive as the rational signs. The uterus may be enlarged in size, we may detect with the finger or speculum, or both, hard tumours more or less circumscribed, but we cannot with any certainty determine whether such tumours are fibrous growths, or indurations consequent on simple inflammation of the womb.

"When we discover," says M. Lisfranc, "one or several tumours, more or less rounded and circumscribed, situated on the womb, it is supposed that those tumours are always fibrous. From this grave error it follows that a merely palliative treatment is adopted, and thus the opportunity is frequently lost of curing tumours, whose progress, because of the inertness of the treatment, remains unchecked, and usually terminate in the death of the patient. It is necessary to prove the truth and value of this opinion, and I shall, therefore, here reproduce the evidence so often adduced at my clinique.

"As inflammation of the uterus may exclusively affect the neck or the fundus of the organ, why may it not also occupy still more limited portions of the womb? and why may it not terminate in indurations more or less rounded and circumscribed?

"We know that inflammation, affecting the thigh for example, may disappear almost completely, only leaving here and there isolated indurations more or less rounded and circumscribed. May not inflammation of the uterus terminate in the same way? Reasoning and analogy favour this view, which is also sanctioned by experience. When I was in the habit of exhibiting operations on the dead body, I have opened the bodies of many women who died of chronic metritis or metro-peritonitis, and frequently observed this to be the fact. Do we not also know that true abscesses, sometimes containing very concrete pus, have been found in the parietes of the uterus? I have met with such an abscess in the centre of a hard tumour, perfectly rounded and circumscribed.

"I have demonstrated round and circumscribed indurations of the uterus, lobulated or uneven on the surface, like fibrous tumours, which they also perfectly resembled in hardness.

"When I commenced practice, I was misled by received opinions, and set down these tumours as fibrous, but I soon discovered that their size in many instances diminished under the influence of antiphlogistics and narcotics, employed with the view of relieving inflammatory and nervous symptoms. I was thence induced to administer discutients when the inflammation had been completely subdued, and thus frequently effected their complete resolution. Need I say, that it is impossible to disperse a fibrous growth? From the foregoing data then I conclude that simple inflammatory indurations of the uterus have often been mistaken for fibrous tumours. I am in possession of numerous cases which bear out this opinion, of which the following are examples." (pp. 17-18.)

The cases here referred to are twenty-six in number, and are given at such length, that they occupy twenty-three pages. It would be superfluous to analyse them. Suffice it to say, they afford examples of indurated tumours of the uterus, closely resembling fibrous tumours, but which yielded to the treatment to be presently mentioned.

"From these observations," says M. Lisfranc, "to which, I repeat, I could add many others, I conclude that there are no certain signs of the existence of fibrous tumours of the uterus previous to their assuming the form of polypi. For the expulsive pains merely raise a strong presumption of the existence of those tumours, inasmuch as those pains may be caused by clots or other foreign bodies in the cavity of the organ, and are sometimes even occasioned by simple tumefaction of the uterus, a fact which I have ascertained by post-mortem examination; the same, as I have already stated, holds good of obstinate and frequently recurring sanguineous discharges, continuing for a long period, and also of augmentation of the bulk of the womb, after its volume has been reduced by appropriate treatment.

"In this uncertainty of diagnosis what course should the practitioner pursue? He should employ the treatment suitable for simple inflammatory induration of the uterus, and by so doing, will frequently succeed in curing the disease. Numerous facts, I cannot too often repeat, sanction this novel opinion, which has proved offensive to many, and has exposed me to numberless attacks.

"Furthermore, fibrous tumours usually cause the same symptoms as simple tumours of the uterus, and it is therefore sound surgery to adopt the same treatment in both cases. If, in fact, we have to deal with a fibrous tumour, we mitigate or even remove the symptoms it excites, and may possibly to a certain extent, prevent their recurrence; finally, we may retard or render stationary its dangerous progress, without obstructing its natural cure by the conversion of the tumour into a true polypus; so far indeed, from the treatment interfering with this salutary result, it, on the contrary, favours its occurrence; if, however, the tumour consists of an hypertrophy either simple or indurated, it has been shown that it may be cured." (pp. 41-3.)

"In some cases, under the influence of the treatment, the apparently fibrous tumour remains stationary, and the patients may live for years, experiencing generally but little inconvenience, though liable to the supervention of severe symptoms which however, usually admit of being remedied. I have met with several cases of this kind, but shall cite only one." (p. 44.)

The history of this case is interesting, inasmuch as the existence of a true fibrous tumour was ultimately ascertained by dissection. During a period of fifteen years, however, its progress was arrested, several intercurrent attacks of subacute inflammation, accompanied with very considerable enlargement and induration of the uterus, were successfully treated, and the patient passed a life of tolerable comfort, until finally carried off by pneumonia.

The treatment pursued by M. Lisfranc in these cases, consists in first dissipating any inflammation or congestion that may be present, by abstinence from sexual intercourse, rest, a non-stimulant regimen, one or more bleedings at the arm to the extent of three or four ounces each, emollient and nearly cold injections into the vagina, laxative enemata, the occasional use of Seltzer water and other mild aperients, and the administration of cicuta, belladonna, and opium, by the mouth or by the rectum, as circumstances may require. When this first indication has been fulfilled, iodine is to be administered in the form of frictions with iodide of lead ointment on the groin, either alone or conjointly or alternately with iodide of potassium internally, and sometimes an issue or seton is inserted in the region of the uterus, or of the ovaries. This of course is a mere outline of the treatment, which must be suspended, resumed, and varied according to the progress of the case. Unwearied perseverance is essential. In one of the cases, the patient was under treatment for thirty-seven months, in the others, a cure was effected in seven, ten, five, eight, and ten months respectively.

As to M. Lisfranc's claim to priority in advancing the foregoing views, it is, so far as we know, partly founded, and partly the reverse. We do not know that any previous author has formally maintained that we can *never* discriminate a fibrous tumour of the uterus, from a simple induration of that organ, though the obscurity of the diagnosis in many instances, and the impossibility of effecting a diagnosis in very many others, is of course well known, and generally admitted. But as to the practice to be adopted in *hard* tumours of the uterus, the French surgeon has been anticipated by Dr. Ashwell, in papers published in 'Guy's Hospital Reports,' seven or eight years since. Dr. Ashwell, indeed, has not enunciated his views with the same precision as the French author; in one place he speaks of *hard* tumours, and then again of *fibrous* tumours. M. Lisfranc, it will be observed, considers the fact of a tumour being absorbed as satisfactory proof that it was not a fibrous growth, an opinion in which we most fully concur. Some English authors, on the contrary, admit the possibility of the absorption of fibrous tumours, but what Dr. Ashwell's opinion on this point is does not very clearly appear. In one place he says, "in hard tumours of the walls or cavity of the uterus, resolution or disappearance *is scarcely to be expected;* since the growths are adventitious or parasitic, and are not imbedded in glandular structure. Here the prevention of further deposit—in other words, the restraint of the lesion within its present limits, and the improvement of the general health—will be the extent of the benefit derived." (Guy's Hospital Reports, 1835, and Practical Treatise on the Diseases peculiar to Women, part ii, 1843, p. 293.) But in two other passages, he informs us, first, that "a hard or fibrous tumour has once, in my own practice, disappeared without the use of any medicine," and secondly that he cannot "think it impossible that this same agent (iodine) may produce absorption." (Practical Treatise, &c. pp. 297-9.) Moreover, Dr. Ashwell (*id. opus*, p. 370) includes within the category of *hard* tumours scirrhous tumours of the uterus, as to the curability of which, we are utterly incredulous. We might readily extend these remarks, but we shall have occasion to notice more fully Dr. Ashwell's labours in this and other respects, when his practical treatise on female diseases shall have been completed, and in the meantime, our object is merely to observe, that, despite of much vagueness and want of precision, he has anticipated, at least in print, the practical conclusions of M. Lisfranc.

*Polypus of the uterus.* M. Lisfranc admits, with M. Malgaigne, five varieties of uterine polypus. The *vesicular*—the *cellulo-vascular*—that consisting of *hypertrophy of the tissue of the uterus*—the *moliform*—and the *fibrous*. Nothing can exceed the tedious prolixity, the fatiguing repetitions, and the defective arrangement of this chapter; but on that very account, and as it must be interesting to know the views of a surgeon of such celebrity as M. Lisfranc, respecting so important a subject, we shall collect into somewhat of a connected form the scattered fragments relating to perhaps every point of sufficient prominence to require notice.

Though the account given of the pathological anatomy of fibrous polypi of the uterus contains scarcely a new fact—does not even give a full view of the state of knowledge on the subject, it will yet be convenient to give a brief abstract—not indeed of all that is said respecting the structure of those tumours but merely—of the statements relating to certain points which, with a few exceptions we select,—not we repeat, because of their novelty, but because they directly bear on certain practical points afterwards adverted to by the author.

Fibrous polypi are covered by an *envelope*, which varies in structure; sometimes it seems to be derived from the mucous membrane of the uterus. Its thickness varies, but it is generally thinner and more adherent at the base of the tumour, and is elsewhere more loosely attached, occasionally so much so, that enucleation can be effected with facility. It may be white, smooth, polished, apparently fibrous, and without a trace of vessels; or, on the contrary, red, rugous, and presenting on its surface a vascular network interspersed with prominent veins, and containing arteries of some magnitude. The envelope very generally alone forms the peduncle of the tumour, and it is not unfrequently perforated or absorbed, so as to expose the polypus at one or several points, and in a few rare cases, M. Lisfranc has seen it completely removed almost to the point of attachment to the tumour. In other cases the envelope is exclusively formed by a large layer of the tissue of the uterus, which has been protruded before the tumour during its progress towards or into the vagina, and then the peduncle consists of the proper substance of the womb; in this case also, the envelope is often perforated to a greater or lesser extent by the included fibrous body; or again, these two structures may be combined, as the investing layer of the uterus may be covered by the preternaturally vascular and thickened lining membrane of the organ. (pp. 58-9.)

Fibrous polypi occur in one of three states. 1st. *Flesby*—when they are either red and soft like muscle, or firmer and paler, perhaps yellowish gray or even white. Their tissue consists of a mass of interlaced fibres, presenting here and there indurated points, probably formed by a more compact interlacement of the fibres. The consistence of the tumour may be very soft, and it may contain distinct blood-vessels as well as a considerable quantity of loose cellular tissue placed between the fibres by which it is essentially formed. 2dly. The tumour may be more or less completely converted into *fibro-cartilage*, a change which commences in the more indurated points above mentioned, and may gradually invade the entire mass. And 3dly. The fibrous growth may become partially or entirely *ossified*. (pp. 61-2.) But polypi, properly so called, scarcely ever undergo this bony degeneration, which is almost peculiar to fibrous tumours, while impacted in the cavity of the parietes of the uterus. When uterine calculi occur loose in the organ, M. Lisfranc is of opinion that they never were pedunculated, as, in three dissections, he found such tumours projecting partially and quite denuded into the cavity of the womb,—in the first stage of their escape in fact; for had the process been continued they must of course have soon become detached. (p. 82.) In one instance however, the only case of the kind M. Lisfranc is aware of, he removed a pedunculated polypus about the size of a hen's egg, and apparently of the usual consistence and density of such tumours, but on dissection, a bony concretion, lodged in a kind of smooth polished cyst, was found within an envelope formed by the tissue of the uterus. (pp. 117-18.) With reference to the once much disputed point, whether fibrous polypi are ever supplied by distinct blood-vessels communicating with those of the uterus, M. Lisfranc adds another case to those already known, which determine the question in the affirmative. (pp. 63, 72.)

Whatever the original condition of a polypus may have been, its structure may be found materially changed by the morbid alterations to which those tumours are liable; and there is considerable difference of opinion as to what those alterations may be. Some maintain that fibrous polypi rarely, or even never, inflame, ulcerate, or suppurate, or at least that those changes are confined to the vascular covering of the tumour; but M. Lisfranc, in common with some previous observers, Dupuytren, for example, has *several times* found abscesses in the very substance of the tumour. (p. 63.) Inflammation may cause polypi to adhere to the neighbouring parts, e. g., to the neck of the uterus, and thus a communication may be established between the uterine vessels and those of the polypus (p. 81); or if two or more polypi coexist, they may, from the same cause become adherent to each other, and give the appearance of tumour with several peduncles. (p.78.) M. Lisfranc has also repeatedly seen fibrous polypi undergo cancerous degeneration, and observes, that the notorious difference of opinion respecting this point "might well excite astonishment, were we not aware how frequently accident influences individual experience, a circumstance very liable to impede the progress of knowledge, unless we are cautious not to deny the reality of facts, merely because they have not happened to fall under our own observation." Occasionally fibrous polypi become infiltrated with fluid, soft, pulpy, and easily broken up. This alteration, which may materially influence the treatment, may result, M. Lisfranc conceives, either from the neck of the uterus constricting the peduncle, and causing congestion of blood in the tumour, or simply from an alteration of its vital properties. (p. 63.)

Some, for example, Cruveilhier, maintain that a fibrous tumour cannot become pedunculated, while it is within the cavity of the uterus, as the parietes of the organ then compress it equally on every side, but that the moment the resistance of the cervix uteri is overcome, a peduncle is formed. (Anat. Path. Livr. xiii.) The inaccuracy of this opinion has been shown by the observation of several cases in which intra-uterine polypi were perfectly pedunculated; and M. Lisfranc adds two additional examples of this to those already known. In dissecting two women, he discovered two large fibrous polypi, both completely incarcerated in the uterus, and both attached by a very *slender* peduncle. This also disproves the notion that the peduncle is formed by the constriction exerted by the cervix uteri. (pp. 73, 150.) M. Lisfranc, however, admits that the pressure of the neck of the uterus causes the deep circular depressions occasionally met with on polypi, and which, on a superficial examination, might seriously embarrass the diagnosis, as the portion of the tumour above the depression, especially when it is very deep, might be mistaken for the uterus. (pp. 61-73.) When a fibrous tumour has passed the orifice of the uterus, it very generally assumes the shape of a polypus, but the author has seen several cases in which the tumour projected a considerable distance into the vagina, without being in the slightest degree pedunculated; in such a case, enucleation may occasionally be very easily effected. (p. 74.)

It is generally admitted that polypi are more frequently connected with the body than with the neck of the uterus; M. Lisfranc has, on the contrary, found even fibrous polypi much more commonly attached to the latter part of the organ. Polypi of the uterus may grow from any point of its internal surface, but, according to Boyer, they are usually inserted somewhere between the orifices of the fallopian tubes, an opinion, which is confirmed by M. Lisfranc's experience, as the tumour occupied this situation in forty-three out of sixty cases, which he either operated on or dissected. (pp. 85-6.)

Formerly, polypus of the uterus was thought to be a very rare disease; M. Lisfranc thinks its frequency is, on the other hand, now greatly overrated, and founds this opinion on the result both of his dissections and of his examination of the living. A large number of females affected with diseases of the uterus attend as out-patients at La Pitié, and from twenty-five to thirty women labouring under similar maladies are constantly inmates of the hospital; his private practice in this class of cases, is also very extensive; and yet during the last fifteen years, he has only seen, on an average, twenty cases of polypus of the uterus yearly. This statement, if it could in any way bear on the question of the frequency of uterine polypi, which obviously it cannot, would disprove the very point our author is attempting to establish, for so far as we are aware, it enormously exceeds what has fallen within the experience of any other practitioner; for example, it is *only double* the number of cases that fell to the share of Dupuytren. (Leçons Orales, t. iii, p. 450.) M. Lisfranc confesses that he has not "dressé une statistique" of his post-mortem examinations bearing on this point, and we suspect he has made the same omission with respect to his living patients. (p. 66.)

In treating of the symptoms and diagnosis of polypi of the uterus, M. Lisfranc especially insists on the following points as being both novel and of practical importance. Thin, narrow, riband-like polypi, and also those of small size, with a long filiform peduncle, are difficult to be detected, as they offer scarcely any resistance to the finger, and consequently communicate to it a very obscure sensation; but by prolonging the examination, the polypus may at length be fixed against the neck of the womb, and its presence be detected by gentle lateral motions of the finger. If the speculum is applied, the riband-like polypus may readily escape observation, in consequence of being glued, as it were, by glairy mucus to the neck of the uterus, but by carefully wiping the parts with a dossil of lint, the morbid growth is detached from its temporary adhesion and brought into view. The existence of a very small polypus with a filiform peduncle, may have been satisfactorily ascertained, both by the finger and by the speculum, and yet on a subsequent examination it may be quite impossible to discover it, as tumours of this kind occasionally reascend into the uterus, and become for a time, inaccessible, because of the orifice of that organ contracting; or if, on the other hand, the neck of the uterus is much dilated, the tumour may be pushed through it before the finger, without being felt. In such cases the first diagnosis must not be hastily assumed to have been incorrect; in either case, the tumour will soon descend again, and be detected on repeating the examination, perhaps, after the lapse of several days. (p. 106-14.) The fact that the inferior orifice of the uterus is more dilated during and for a few days before and after menstruation, and that intra-uterine polypi are consequently then most readily detected, is stated amongst the circumstances grouped together as new, (p. 107;) but has been long since specially stated by other authors, e. g. Dupuytren. The same observation applies to several other statements, which we abstain from noticing.

The differential diagnosis between polypus and inversion of the uterus is considered at great length; and the following observations contain everything, we believe, that is said on this head, to which it is necessary to advert. It is very generally said, that a polypus is insensible, and that an inverted uterus is sensible to the touch. The inaccuracy of this distinctive sign is, no doubt, known. Dr. Johnson, for example, mentions a case, in which a polypus was very sensitive to the touch, (Dublin Hosp. Reports, vol. iii,) and Dupuytren says that polypi cease to be painless when their surface becomes inflamed. M. Lisfranc says that he has *very frequently* met with polypi which were painful on pressure, and assigns, we have no doubt, the true explanation of the fact, viz. that the tumour is frequently covered by the proper tissue of the uterus, though even then the lower part of the tumour may be insensible, on account of the envelope having become very thin, or even perforated in that situation. (p. 133.) When a polypus, or an inversion of the uterus, as the case may be, has scarcely dilated the neck of the womb, it is supposed to be impossible to distinguish the two affections.

"If," says M. Lisfranc, "the symptoms were very serious, and the life of the patient in danger, it would be our duty could we ascertain the existence of a polypus, to attempt to remove it; would it not then be allowable to seize the neck of the uterus with Museaux's forceps, and depress it to the vulva? and if on then passing the finger into the rectum, the body of the uterus was felt to be considerably enlarged, might we not conclude that we had to deal with an uterine polypus? . . . . . but, if on the contrary, it was found that the size of the uterus was not increased, that its longitudinal diameter was diminished, would not this indicate that the case was one of inversion? Guided by these new data in a case such as is now under consideration, I succeeded in removing a polypus, and rescued the patient from apparently certain death." (p. 136.)

In partial inversion of the uterus, M. Lisfranc thinks favorably of the mode of examination proposed by M. Malgaigne, which we shall describe, as we do not recollect that it has appeared in this Journal. In this affection, the bladder and a portion of the intestines are lodged in the concavity formed by the depression of the fundus of the uterus; if then, a curved catheter is passed into the bladder with its concavity downwards, and the beak of the instrument is directed to the most depending part of the bladder, its extremity will be readily felt by the finger in the vagina, if the case is one of inversion, unless, indeed, the intestines have become adherent to the womb in such a way as to prevent the catheter penetrating into the depression formed by the inverted organ, a circumstance of very rare occurrence. (p. 137.) But M. Lisfranc thinks that the best way of discriminating between polypus and inversion of the uterus, is by a mode of examination similar to that above recommended, in the case of an intra-uterine polypus or of a commencing inversion. If we seize and depress the tumour with two fingers passed into the vagina, and then introduce the index-finger of the other hand into the rectum, no tumour can be felt through the gut above the one which is grasped in the vagina, if the case is one of inverted uterus. But if, on the contrary, we feel through the rectum a second tumour similar in shape to the uterus, above the vaginal tumour, then this latter tumour is a polypus. In one instance, indeed, M. Lisfranc was misled by this mode of examination; he diagnosticated inversion of the uterus, but the patient having died, a small fibrous tumour was discovered implanted on the uterus, which was flattened and reduced to the tenth part of its natural size. (pp. 139, 174.) It appears that attempts have been made to defraud the author of the honour due to this suggestion, as he subsequently "begs leave to thank the authors who have appropriated his ideas, or with characteristic candour cited them as dating from the eleventh century." (p. 385.) It is not stated who are the delinquents here alluded to, and we are not able to supply the omission.

A great number of circumstances connected with the shape and disposition of the tumour may render the exact nature of a case more or less obscure. Under this head we shall only notice the following case, as we do not recollect having met with an account of one similar to it. A polypus was distinctly felt in the vagina, apparently attached to a peduncle, which could be traced for about half an inch through the neck of the uterus, but its point of insertion could not be reached. The tumour was easily drawn through the vulva, and on then passing the finger along the supposed peduncle, it was found to be attached to and surmounted by, or rather to expand into, a second fibrous tumour. So far the case is merely an example of one of those circular depressions on a polypus, which have been already adverted to. But, on examining the upper portion of the tumour, the finger could be carried fairly round it, without discovering any point at which it adhered to the womb. During the protracted examination that now took place, the uterus was considerably depressed, and at length an adhesion was felt between the inner surface of that organ and the supposed peduncle; on dividing which, the tumour was found to yield, and was soon extracted. The adhesion in fact, which was thought to be accidental, was the peduncle of the tumour. M. Lisfranc supposes that the polypus, during its development, had grown partly towards the vagina, and in part towards the cavity of the uterus. (p. 74.)

When a polypus of the uterus is detected, the indication is to remove it if possible, for with some exceptions the symptoms increase in severity, and a *spontaneous cure* is a very rare event indeed. This interesting point in the natural history of the disease is considered very imperfectly by M. Lisfranc. He mentions two circumstances under which the natural cure may occur: 1st. The peduncle may become elongated and slender, and finally break from the mere weight of the tumour, an event which *he has seen occur in four cases*, where the neck of the uterus was so dilated that it could not have exerted any pressure on the peduncle. (p. 80.) 2d. The pressure of the neck of the uterus on the polypus may act as a ligature, and thus produce a spontaneous cure; this, M. Lisfranc observes, is a very rare occurrence, but he witnessed it in one case which is recorded. (p. 81.) It would thus appear that the author has observed *five* examples of spontaneous cure, but he subsequently informs us, "A spontaneous cure is a very uncertain event. I have only seen *three* cases in which it occurred; a few others have been recorded." (p. 155.) No allusion is made to other modes in which fibrous tumours and polypi have occasionally been naturally gotten rid of, such as their expulsion during parturition, and their separation in consequence of absorption or rupture of the cyst or envelope by which they are covered. Small fibrous polypi occasionally cause but little inconvenience. This is a fact well known; but M. Lisfranc mentions a very remarkable circumstance, that he has had several patients who would not submit to any operation, though they long suffered from attacks of severe uterine hemorrhage; in some of these cases the bleeding disappeared gradually, in others suddenly, and has not returned during three, four, or even five years; the other symptoms underwent a corresponding amelioration, and the patients continue to enjoy tolerable health. (p. 155.)

With respect to the palliative treatment of uterine polypi, we shall only advert to M. Lisfranc's practice in certain cases where hemorrhage has reduced the patient almost to extremity; but we first state his views as to the source of the hemorrhage, as it is on that the practice is based. We shall make no array of the conflicting authorities respecting this point; M. Lisfranc agrees with those who refer the hemorrhage principally to the surface of the tumour. "Some polypi," he says, "do not bleed, and then the very vascular membrane which usually covers them is entirely wanting, and their surface is generally covered *by the tissue of the uterus alone.*" (p. 58.) And again, "The source of hemorrhage is almost always the mucous membrane enveloping the tumour, and only occasionally, in some extremely rare cases, the polypus itself: the uterus, however, may unquestionably be implicated as a cause: 1. Because it is often congested, and congestion is alone sufficient to excite an afflux of blood. 2. Because the irritation of the polypus may produce the same effect, and the womb has been found in a state similar to that which it presents during menstruation." (p. 99.) We shall only remark on these passages, that we think M. Lisfranc under-estimates the importance of the altered condition of the uterus as a cause of hemorrhage, as it is well known that that organ when affected with polypus often becomes hypertrophied, softened, and permeated with vessels resembling the sinuses of the gravid uterus, a condition in which the layer of its tissue *covering* the tumour and forming its peduncle has been distinctly seen to participate; but we shall not dwell on this, as our object is merely to come to the practical application of M. Lisfranc's views. If then a patient labouring under polypus is almost exsanguine and reduced to a state of extreme debility, the exertion and fatigue consequent on an operation may be not exempt from danger, the trifling loss of blood that may follow either excision or the application of a ligature might prove fatal, and though the debilitated condition of the system is unfavorable to the development of inflammation, yet if inflammation were to follow the operation it would probably terminate in death.

"Under such circumstances," says M. Lisfranc, "if the polypus is not too large, if it is covered by the vascular membrane which almost always alone yields the hemorrhage; I tear off that membrane when I can reach it with my fingers, and when it is not too firmly adherent to the subjacent parts; or I apply the largest possible speculum, and cauterize the surface of the tumour freely with the acid protonitrate of mercury; by these means I have always, hitherto, restrained the hemorrhage; and it is superfluous to add, that I operated on the tumour at a subsequent period with a much better chance of success." (pp. 160.)

If a polypus completely within the cavity of the uterus is accompanied with uncontrollable menorrhagia, an operation may appear urgent, but is greatly more difficult and dangerous than when the tumour is in the vagina; in this case the neck of the uterus is very frequently dilated, and M. Lisfranc took advantage of this circumstance in several cases to apply, with the aid of the speculum, protonitrate of mercury to the small accessible portion of the tumour. This practice has almost always succeeded in his hands, especially when repeated twice or thrice within twenty-four hours,—in fact, whenever the bleeding returned. He is inclined to explain the efficacy of cauterizing so very limited a portion of the tumour, by saying that the polypus is compressed by the parietes of the uterus over its entire surface except at the point corresponding to the orifice of the uterus, in which situation consequently it can alone bleed. On removing polypi which had been thus cauterized, a slough was found a few lines deep, and a considerable portion of the tumour was inflamed. M. Lisfranc has never tried this practice except in the case of fibrous tumours, and cannot say how far it might be serviceable with cellulo-vascular and red soft spongy polypi. (pp. 159-67.)

A variety of circumstances may have to be considered before we undertake the removal of a polypus by operation. A polypus may coexist with rounded hard tumours of the uterus, which may be either fibrous or merely the consequence of a long antecedent chronic inflammation; but be their nature what it may, their presence does not contraindicate the removal of the polypus, for if the tumours are not fibrous they will probably disappear even spontaneously, and if they are, though their presence certainly renders the operation more dangerous, yet far greater danger would be incurred by non-interference. (pp. 43, 145.) If a polypus is complicated with diffuse induration of the uterus, occupying perhaps the greater portion of its anterior or posterior surface, as is frequently the case, or even involving the entire organ, the question arises whether we should first treat the polypus or the hypertrophy of the womb, and the answer depends on the presence or absence of chronic inflammation, the existence of which we should always suspect and carefully look for in such cases. If chronic inflammation is present it must be dissipated by appropriate treatment before the polypus can be safely operated on, for if this precaution is neglected an attack of acute inflammation would probably supervene and place the patient in the greatest danger, or at all events symptoms of uterine disease would continue to the distress of the patient and discredit of the practitioner. Nevertheless, if the symptoms are urgent, if the patient is being run down by obstinate menorrhagia, this risk must be encountered and the operation cannot be deferred. If no signs of chronic inflammation can be detected, or when they have been dispelled, the polypus should be at once removed, as its presence irritates and congests the organ. Sometimes the hypertrophy rapidly disappears after the operation; in others it yields slowly, or may even permanently remain. (pp. 145-9, 158, 176, 219, 228.)

Ulcers of the neck of the uterus are often completely masked by a polypus, and the surgeon should therefore be cautious in promising speedy and complete relief from uterine distress as the result of an operation. Many are of opinion that the vaginal discharge diminishes but does not entirely cease for some time after the removal of a polypus, M. Lisfranc has almost invariably observed that the discharge ceases promptly and completely, unless the neck of the uterus was ulcerated or the organ was obviously enlarged. (144-8.)

Anemia, extreme debility, emaciation, colliquative diarrhea, &c., do not contra-indicate an operation provided no organic disease of any important viscus can be detected. (p. 147.) On this last point, especially as regards disease of the lungs, the author lays peculiar stress; he states that he has *frequently* seen females affected with tubercles of the lungs, in whom, on the appearance of a polypus of the uterus, with its accompanying irritation and discharge, the progress of the thoracic disease was so completely suspended, and its symptoms so much amended that the patient forgot, as it were, that it had ever existed, and the surgeon could not even suspect its existence without a most careful inquiry into the history of the case, and a scrupulous examination of the thorax. In such cases, if the symptoms caused by the polypus are mild and not progressive, they must not be interfered with, as experience has shown that if we unfortunately succeed in mitigating them to any considerable extent, the symptoms of phthisis supervene and soon prove fatal. But if menorrhagia, ulceration, or hypertrophy endanger life, our object should be to ameliorate the symptoms, but not to remove them, nor even, if possible to materially mitigate them. M. Lisfranc states that he has seen the lives of "*a great number*" of patients prolonged by such management (*une thèrapeutique de bascule*) for four or even six years. (p. 230.)

The selection of the period for operating on a polypus is of great importance. Useless delay would be dangerous, we should not operate within ten days before or eight after the period of menstruation; M. Lisfranc has several times witnessed serious and even fatal consequences from the neglect of this rule. (p. 219.)

Polypi situated entirely within the cavity of the uterus have been successfully removed by Herbiniaux, Desault, Deguire, Dupuytren, Recamier, and others, but as they generally make their way into the vagina, that event should be waited for, unless the circumstances of the case imperatively require interference. If the incarcerated tumour has become soft and pulpy, it may be removed by *avulsion* (*arachement*), and this is the only case in which M. Lisfranc recommends this mode of operation: what he terms *avulsion* is indeed a combination of that process with *laceration* (broiement). He thus succeeded in breaking up two intra-uterine polypi, and having removed as much of the mass as was practicable, left the rest to slough away. (pp. 63, 64-8.) In one instance he unintentionally removed a polypus by true avulsion. While attempting to depress the tumour for the purpose of excising it, a sound, as of something giving way, was heard, and the polypus was found completely detached. (p. 39.)

M. Lisfranc has on several occasions removed by *enucleation* both polypi and fibrous tumours which were not pedunculated, whether situated completely within the cavity of the uterus, or having partly (or in the case of polypi entirely) made their way into the vagina. To use his own words, he "dwells on this important point of practice which he believes to be new." (p. 177.) We need not occupy space in showing that the practice is not new, but as we believe M. Lisfranc has adopted it with more boldness than his predecessors, and under circumstances in which it was not previously applied, we shall give a summary of a few of the cases by which he illustrates this practice.

In one case having drawn a fibrous polypus almost entirely through the vulva, he perceived that its envelope, which consisted of a thin layer of the tissue of the uterus, was lacerated, and passing the index-finger through the rent, enucleated the tumour with the greatest facility. (p. 172.) In another case enucleation was effected almost accidentally: M. Lisfranc, while examining a polypus, found the envelope give way beneath the nail of the index-finger, and by an easy manipulation enucleated the tumour in a few seconds. On examining the uterus immediately afterwards, he found that the part of that organ to which the polypus had been attached, had singularly contracted, that the depression caused by the tumour had diminished greatly in depth, and at least two thirds in breadth, it seemed to be diminishing while the finger was in contact with it, and in ten hours the uterus had regained its natural size, and the cervix would not admit the finger. (p. 173.) We mention these latter facts, as we conceive they have an important bearing on the question of hemorrhage after excision of polypi. M. Lisfranc has also frequently enucleated with the nail of the index-finger, small cellulo-vascular polypi occupying the neck of the womb. (p. 176.) In a case where a fibrous tumour as large as the clenched hand projected into the vagina, its envelope was lacerated with the nails, and the contained tumour turned out. (p. 73.) But enucleation must generally be preceded by an exploratory incision; and by this combination of means, M. Lisfranc has removed fibrous tumours while still completely included within the cavity of the uterus. A lady was reduced almost to extremity, by protracted uterine hemorrhage caused by a fibrous tumour, which could be felt through the dilated cervix uteri. The neck of the uterus was seized with Museaux's hook, depressed almost to the vulva, and a more perfect examination being then practicable, the tumour was found to extend from the middle of the body of the uterus almost to its lower extremity, and to be lodged in its posterior wall, from which it was commencing to disengage itself. With a straight blunt-pointed bistoury passed along the forefinger, a vertical incision was slowly and cautiously made over the tumour until the finger was enabled to be insinuated beneath the envelope and complete the enucleation, which was not accomplished without some difficulty. Occasionally enucleation may be more easily achieved by substituting a spatula for the finger. (p. 179.) If it is necessary to enlarge the incision in order to effect the removal of the tumour, a grooved director will often guide the knife more conveniently and safely than the finger. In some cases, where the cervix uteri was insufficiently dilated, M. Lisfranc divided it anteriorly. (p. 181.) Whenever the peduncle of a polypus is very broad, we should incise the envelope, and endeavour to enucleate the tumour; in this, however, we cannot always succeed. If the tumour is removed, the envelope sometimes contracts and cicatrizes, sometimes sloughs in whole or in part. (pp. 211-14.)

*Ligature*. We need scarcely say M. Lisfranc rejects this, in common with almost all French surgeons, as a general mode of removing uterine polypi. In this opinion we altogether coincide, and confess our inability to understand the preference given to this method by many respectable British practitioners. It would be out of place to recapitulate the well-known and often repeated objections to the use of the ligature as compared with the operation by excision, as M. Lisfranc adds nothing to the perfectly conclusive arguments long since adduced in favour of the latter proceeding. In certain cases, however, the ligature should be employed: thus, if a polypus of moderate size is completely included within the uterus, and is implanted high up, especially at the summit of the organ, and if the symptoms imperatively demand an operation, a ligature should be applied *if its application is possible*, as it occasionally is when a sufficiently small peduncle can be detected. (p. 217.) Or if a patient is so exsanguine, that the smallest loss of blood is to be dreaded, we should employ the ligature unless the peduncle is too thick, or unless we are unable to bring it when bulky, fairly within our reach, and pierce it with several needles, each armed with a double ligature, and thus tie it in two or more separate portions. (pp. 192-218.) M. Lisfranc disapproves of excising the polypus below the ligature immediately after its application, as we may be compelled to remove it in consequence of its producing serious symptoms, but when the vitality of the tumour is destroyed, as much as possible of it should be cut away. (p. 213-4.)

How should we proceed if an artery is felt pulsating in the peduncle of a polypus? This question has been considered by Dupuytren, and is now discussed by M. Lisfranc, but both authors apparently deal with it merely as a matter of speculation, as neither appear to have met with a case of the kind. Dupuytren recommends excision, having previously applied a ligature of reserve to be tightened in the event of hemorrhage occurring. To this M. Lisfranc objects that the precaution would be illusory, as hemorrhage sometimes supervenes several hours after excision, (p. 214,) and that as the ligature of reserve must of course be left loose, it would slip off were the peduncle very short, or if the peduncle, however long, consisted of the tissue of the uterus, it would probably retract to such an extent, as to escape from the ligature, a circumstance sufficiently exemplified in what has been said respecting enucleation. (p. 217.) In the case now under consideration, M. Lisfranc, if he determined to use a ligature, would at once tighten it so as to thoroughly strangulate the peduncle, then excise the polypus, and eight or ten hours subsequently remove the ligature. Hemorrhage, he is satisfied, need not be then apprehended, for in amputation of the neck of the uterus, the uterine arteries are often opened, and he has frequently restrained the consequent hemorrhage by plugging, but never found the bleeding return on removing the plug after five or six hours; and he has no doubt that such would be the case were an artery in the peduncle of a polypus divided. (p. 214.) If the artery felt in the peduncle was not large, M. Lisfranc would at once perform excision, being perfectly convinced that plugging would certainly control hemorrhage, should it supervene. (p. 216.)

In removing a polypus either by *ligature* or by *excision*, it has been much debated whether it is necessary to divide the peduncle close to the uterus, in order to prevent a return of the disease. Levret and many subsequent writers assert that the entire of the peduncle dies, no matter at what point it may have been tied. Dubois, on the contrary, is of opinion that the portion of the peduncle above the ligature dies only when the ligature is placed near its point of implantation, and that it lives when tied low down. Dupuytren too insists on the necessity of cutting the peduncle as close as possible to its insertion, as he is of opinion that any portion of it which may have been left, is very likely to grow again. This opinion, it is clear, Dupuytren founded on an erroneous view of the pathology of the disease; for we conceive that the peduncle always contains fibrous tissue, that the globular portion of the tumour in fact is connected to the womb by means of a slender fibrous stem, occupying the centre of the peduncle, a circumstance which, though it sometimes occurs, is very rare—is in truth not the rule but the exception. M. Lisfranc has occasionally been compelled to tie polypi at the centre of their longitudinal diameter, and found that the *whole* of the tumour died. In one instance, an enormous polypus filled the vagina, there was a deep circular depression at the centre of the tumour, round which a ligature was passed, in the expectation that when the lower part of the mass was removed, the residue would be more easily dealt with, but the portion above the ligature sloughed as well as that situated below it. (pp. 189-90.) He also witnessed several other similar cases disproving Dubois's opinion. We also find a case in which an attempt was made to excise an intra-uterine polypus, but the peduncle could not be detected, and a portion of the tumour, estimated at about half its bulk, was cut away; the part left behind, however, sloughed, and the patient recovered perfectly. (pp. 242-8.) It cannot, however, be denied that the tumour occasionally grows again when a portion of the peduncle has been left, and this difference of result in different cases cannot be foreseen or explained in the present state of our knowledge. (p. 212.)

As to the after-consequences of operations on uterine polypi, bad symptoms seldom supervene unless a *ligature* or *caustic* has been employed; and from the cessation of the discharges, the relief from suffering, the return of appetite, &c. the patient imagines she is perfectly restored to health, an opinion in which the surgeon unfortunately too often coincides. This impression, if prematurely acted on, may lead to disastrous consequences, because a slight degree of sub-acute inflammation may latently exist, or even if it does not, yet metritis may be readily excited by early indulgence in the most moderate exercise, by any error of diet, by a chill, &c. M. Lisfranc consequently recommends that absolute rest in the horizontal posture should be observed for three weeks after operation, that the diet should be cautiously regulated for some time, and that the patient should be enjoined to resume her ordinary habits very gradually and cautiously. (pp. 219-22.)

*Hemorrhage* after excision is scarcely to be apprehended. M. Lisfranc, after referring to Dupuytren's experience of the rarity of its occurrence, states that he has himself performed the operation 165 times, and that hemorrhage followed in but two cases. In one case scarcely a drop of blood flowed at first, but in three hours violent bleeding came on, and was restrained by plugging; in the other the hemorrhage was not alarming until five hours after the operation, and was similarly restrained; in both the plug was removed on the evening of the day of the operation without any recurrence of the bleeding. (p. 219.) Not unfrequently a slight sanguineous discharge occurs either immediately after, or one or two days after the operation. It usually comes from the wound inflicted, but may come from an ulcer or from a second polypus that escaped detection. If the patient is not very weak, and if there is no pain, &c., it is well to let this discharge continue for some days, as the patient is thereby gradually accustomed to the cessation of an habitual hemorrhage, and congestion of the uterus may be relieved if it exists, or warded off if it does not. But if the slight flow of blood continues five or six days, or if it is accompanied with pain, shivering, heat of skin, or a sense of weight in the pelvis, interference is requisite, as congestion of the womb exists, is becoming aggravated, and may terminate in inflammation, to prevent which we should adopt the treatment indicated for menorrhagia. If the hemorrhage is caused by a second polypus, by hypertrophy, or by ulceration of the polypus, it may come on unexpectedly when the patient seems almost completely recovered. In the first case it is very obstinate; in the second it usually yields very readily to treatment; in the third, cauterization at once restrains it unless the disease is very extensive. (pp. 125-6.) When no hemorrhage follows the operation, as is usually the case, the sudden suppression of the habitual drain predisposes the patient to visceral congestions and inflammations, and in any case when health has been reestablished, the symptoms of general plethora and of local congestion should be carefully watched for, and combated by venesection and an appropriate regimen. The return of natural menstruation does not sufficiently guarantee the patients from those mischiefs, and they are still more to be apprehended when menstruation is scanty or suppressed. (pp. 229-31.)

The wound caused either by ligature or excision generally heals rapidly, but sometimes remains ulcerated for a long time—for upwards of a year, as M. Lisfranc has witnessed not unfrequently. The wound, or rather ulcer, may then cause congestion of the womb and leucorrhea, or it may even degenerate into cancer. It is therefore necessary sedulously to examine the progress of the wound caused by those operations. (pp. 193, 201, 228.)

Hypertrophy of the uterus, if not indurated, frequently disappears spontaneously in twenty or thirty days after the removal of a polypus. If the tumefaction is hard, but recent and of slight extent, it also usually yields under simple attention to the general health. But occasionally M. Lisfranc has seen indurations, apparently of the simplest kind, become aggravated, and even terminate fatally, and he thence inculcates the necessity of carefully watching those cases, and attempting to disperse the induration by treatment, if it shows no disposition to disappear spontaneously. The surgeon should never tell his patient "you are cured," until after he has thoroughly examined the condition of the uterus. (p. 227.)

Some females after the cure of a polypus, or of any other disease of the uterus, fall into bad health, without any viscus being affected, and without the functions of the uterus being at all deranged, and yet general treatment fails to restore health. Under such circumstances M. Lisfranc thinks an issue in the arm indispensable. Some prefer inserting the issue in the leg or thigh, but M. Lisfranc is satisfied, from repeated observations, that the issue is there positively injurious, as it excites an irritation and congestion of the lower extremity, which solicits a flow of blood to and causes congestion of the uterus. (p. 233.)

*Polypi of the vulva.* M. Lisfranc has frequently seen tumefaction or prolapsus of the mucous membrane of the urethra, mistaken for polypus, but on compressing the tumour between the finger and thumb, it is generally much diminished in size, and can often be returned into the urethra, and if a catheter is then passed into the bladder, no foreign body, and sometimes even no thickening of the parietes of the urethra can be felt. If pressure fails to reduce the tumour, it is sometimes, at least to a considerable extent, returned by passing a large catheter. The protruded mucous membrane may become indurated, and the diagnosis is then beset with difficulty, but this is of little importance as the tumour must be removed, if it cannot be returned. If the protrusion can be reduced, scarifying the internal surface of the urethra is often beneficial, but the best mode of treatment when the tumour is not indurated or affected with acute inflammation, is to touch it lightly with nitrate of silver every fifth or sixth day. Polypi of the vulva are seldom fibrous; they are sometimes mucous or cellulo-vascular, but are more commonly red, and similar to those termed "*vivaces*" by Levret. They should be removed with the knife and not by ligature, as they are seldom superficially attached, but almost always extend into the subjacent parts to a considerable depth, and are consequently apt to return when removed by ligature; but M. Lisfranc has never known a relapse occur when they were excised, and care was taken carefully to dissect out their ultimate ramifications. (pp. 248-54.)

*Polypi of the vagina.* These growths resemble in structure those of the vulva. They are much less likely to bleed than uterine polypi. If at all bulky, they usually cause partial inversion of the vagina, in which a portion of the rectum or of the bladder may be included, and apparently constitute the peduncle of the tumour, and consequently either of those organs might be opened, if the surgeon, while operating, did not bear this important fact in mind, and guide his incision by passing into the pouch the finger in the rectum, or a catheter in the bladder, as the case may be. When superficially attached to the mucous membrane they are easily removed, but when more deeply connected, they are liable to relapse unless thoroughly extirpated, a proceeding which endangers the rectum or the bladder. The ligature is objectionable for the reason assigned in the last paragraph, and also because the vesico-vaginal, or recto-vaginal septum, might be included in it. (pp. 255-64.)

*Tumours in the recto-vaginal or vesico-vaginal septum.* These tumours have not been much investigated. They may be situated between the vagina and the rectum, or between the vagina and the bladder, or they may occupy the thickness of the parietes of any one of these organs. The vagina covering them may be either thickened, or the reverse. In one instance M. Lisfranc states that the tumour had perforated and projected into the rectum, and in another it had partly made its way through the anterior wall of the vagina. M. Lisfranc has very seldom seen tumours *between* the vagina and the bladder; he has met with eleven *between* the rectum and the vagina, *three* of which were fibrous, *two* were enlarged lymphatic glands, *four* were simple white indurations, the result of subacute inflammation, and two were scirrhous. Tumours occupying the proper coats of the vagina or of the rectum are much more common; but M. Lisfranc has never seen a fibrous tumour in these situations. The existence of one of these tumours is easily detected, but it may be difficult or impossible to determine its nature. The treatment, however, should always be directed to effect its restoration; and if this cannot be effected and the tumour is very painful, the author recommends that an exploratory incision should be made over it, provided it is rounded and circumscribed. He has thus succeeded in enucleating two fibrous tumours. It may be objected, that the growth may be scirrhous, and if so would be thus converted into open cancer. To this M. Lisfranc replies, that he only advises the practice when the tumour is painful and the symptoms cannot be relieved, and that there is a chance of meeting with a fibrous tumour.

Occasionally the distress in those cases is caused by the pressure of the cervix uteri on the tumour. A lady suffered the most intolerable pain, radiating from the pelvis to the loins and down the thighs, whenever she attempted to take exercise. Anteversion with prolapsus of the uterus was discovered, and a pessary was applied, but could not be borne for a moment. M. Gensoul was now consulted, and detected in the posterior wall of the vagina a tumour the size of an almond, and exquisitely sensitive on pressure; he applied the pessary "*en poire aplatie*" of M. Mayor of Lausanne, which, from its peculiar shape, rectified the position of the uterus without exerting any pressure on the tumour. While this instrument was worn the patient could walk with comfort, but when it was removed, her sufferings returned. In a second case of M. Gensoul's, relief was obtained by the use of an ordinary pessary. And in two patients under M. Lisfranc's care the same end could only be achieved by the application of M. Mayor's instrument. (pp. 264-75.)

*Moles.* A mole is defined by M. Lisfranc to be "a fleshy, organized, insensible mass, usually soft, but sometimes more or less hard; its shape is exceedingly variable; it is formed and developed within the womb, from which it is generally supposed to be expelled a longer or shorter period after its formation." (p. 275.) Many authors consider that moles must necessarily be products of conception; but M. Lisfranc coincides with those who maintain that this is by no means uniformly the case. If the embryo dies, it may become extremely atrophied, or even disappear completely; while the placenta, which still retains its connexion with the uterus, may increase and become altered in structure. M. Lisfranc has no doubt that moles are sometimes thus formed, as he has dissected several wombs which contained placenta in which not a vestige of an embryo could be discovered. (p. 281.) But it is indisputable that moles may be formed independently of conception or sexual connexion, and M. Lisfranc then attributes their origin to coagula of blood which have become organized. (pp. 120-1, 282.) Thus the author has seen females long affected with menorrhagia, during which they abstained from sexual intercourse, subsequently void moles; and on dissecting a young girl who died from the consequences of imperforate vagina, he discovered a mole in the uterus. (pp. 282-3.) But few cases are known in which more than one mole existed in the uterus: M. Lisfranc gives a case in which two moles were expelled within half an hour. (pp. 280-90.) M. Lisfranc discusses at considerable length the various circumstances that have been supposed to distinguish moles from pregnancy, and comes to the conclusion that the diagnosis of the former affection is difficult, or, more properly speaking, impossible; the utmost that can be done is to calculate probabilities, after having carefully weighed all the symptoms. (pp. 292-9.) The period that moles remain in the uterus is very uncertain, but they are usually expelled after from sixty to ninety days, though sometimes not for six or seven or even eight months. Their expulsion is often difficult, attended with as much or even more suffering than parturition, and accompanied and followed by hemorrhage. The hemorrhage is sometimes extreme, so that life would be perilled if the surgeon did not promptly extract the foreign body. When the mole has been gotten rid of the lochia flow and the breasts swell, and are supplied with milk usually just as after labour. (pp. 299-301.) As to treatment, moles should not be interfered with so long as they occasion no bad symptoms. If hemorrhage occurs while the mass is still within the uterus, and the neck of that organ is not dilated, and if the means ordinarily adopted fail to restrain the bleeding, so that life is endangered, the hand should be passed into the uterus, even if a certain degree of violence is requisite in so doing; for, however dangerous this resource, it presents a chance of safety. Incision of the neck of the uterus is imperatively requisite if adhesions exist, or if it is impossible to pass the hand or a suitable instrument into the womb. (p. 307.)

The chapter on "*coagula of blood in the uterus*" (pp. 309-17) contains nothing necessary to be here adverted to.

*Physometra or tympanitis of the uterus.* This disease, it is said, may arise from three causes: distension of the womb from the entrance of atmospheric air; dilatation of the organ by the elastic fluids (sulphurated hydrogen, it is supposed,) generated during the decomposition of organic matter, such as the placenta, a fetus, false membranes, coagulated blood, &c.; or, finally, expansion due to a secretion of gas from the internal surface of the uterus. Whatever may be the source of the elastic fluid, it must be assumed that the cervix uteri is occluded, whether by spasm, a clot, inspissated mucus, a false membrane, a polypus, &c.; or from displacement of the uterus bringing the orifice of the cervix in close and direct apposition with the vagina. It will, we believe, scarcely be denied that gas may accumulate in the uterus in consequence of the putrefaction of a fetus; but the existence of what we may term *dynamic* physometra has been questioned on high authority. Though Frank, Mauriceau, and Delamotte have recorded cases in which the disease was *dynamic* or caused by a morbid secretion of air, yet, as M. Lisfranc says, he has "in this chapter treated of a disease the existence of which is denied by some physicians." M. Lisfranc does not name the physicians to whom he alludes; but we conjecture that he refers to MM. Stoltz and Naegele, two of the most celebrated practitioners in the diseases of females of the present day, who, at the medical congress held at Strasbourg September 1842, expressed their belief that tympanitis of the uterus was impossible, and that the alleged cases of its occurrence were apocryphal. M. Lisfranc has seen several cases in which physometra was caused by the decomposition of extraneous matter included in the uterus, (p. 229;) but only one of these cases is stated. In it the womb extended three inches above the pubis, and occupied nearly the whole transverse diameter of the hypogastrium; on examining the uterus with the finger in vagina, the other being applied on the hypogastrium, he felt a tumour of extraordinary elasticity; during the manipulation the neck of the uterus suddenly dilated, a volume of gas escaped with considerable noise, and the abdomen resumed its natural size; the uterus, however, remained slightly dilated, and at short intervals audibly expelled portions of air; after the lapse of a few days a fleshy mole was expelled. (pp. 321-2.) M. Lisfranc argues, that as the mucous membrane of the intestinal canal indisputably often secretes air, it is unreasonable to deny that the lining membrane of the uterus may also do the same; and in answer to the objection, that any gas generated in the womb when its cervix is not mechanically obstructed must escape, he observes, that every surgeon who has had much experience in examining the uterus must have often observed the remarkable facility with which the inferior orifice of the uterus contracts. We must, however, observe, that M. Lisfranc does not appear to have actually witnessed any case in which uterine tympanitis was purely *dynamic*, that is, unconnected with the presence of any extraneous substance in the womb; we therefore are induced to refer to a case recorded by M. Tessier, (Gazette Médicale de Paris, 5 Janvier, 1844,) which has fallen under our notice while writing this article. A woman aged forty-three suspected that she was pregnant, in consequence of suppressed menstruation, coinciding with a gradual increase of the size of the abdomen, for at least six months; by which time the uterus had ascended almost to the umbilicus. M. Tessier diagnosticated tympanitis of the uterus, because of the remarkably clear sound yielded by the abdomen on percussion, the absence of the stethoscopic sounds peculiar to pregnancy, and the lightness of the tumour formed by the uterus. The patient was at length attacked with pains similar to those of labour, which were soon followed by the expulsion of a large quantity of fetid gas, with relief of all the symptoms; "no clot, no mole, nothing which could refer the origin of the gas to the putrefaction of any foreign body included in the uterus was expelled. Three years have elapsed since the first expulsion of gas, and though the patient has not since been pregnant, the abdomen has frequently become swollen, and again in a few days subsided after the expulsion of a certain quantity of gas." M. Lisfranc does not absolutely deny the possibility of the uterus being distended by the entrance of atmospheric air; but, looking to the structure of the organ, he is strongly inclined to doubt that the affection can ever occur from this cause, except when the womb has been previously amplified beyond its usual dimensions, as, for example, after pregnancy. As to the treatment of the affection, it must obviously be modified according to its exciting causes; and we find but two points necessary to be adverted to. In this, as in every other uterine disease, M. Lisfranc strenuously objects to the employment of intra-uterine injections, inasmuch as they are, however bland the fluid may be, calculated to excite metritis or metro-peritonitis; the recommendation to promote the expulsion of the gas by efforts on the part of the patient he also considers very injudicious; but, with these exceptions, the practice recommended is similar to that usually inculcated. (pp. 317-30.)

*Noisy expulsion of gas contained in the vagina* we find designated by the uncouth phrase, *œdopsophie.* This disease cannot occur when the inferior orifice of the vagina is largely open; but if it is obstructed by inflammation, or is naturally very narrow, gas may accumulate in the canal, just as injections are often long retained in it, though the patient stands or even walks. The gas may be generated by putrescent matter, may pass from the rectum when a fistula exists, may be simply atmospheric air, which readily finds entrance when the lower portions of the labia, are drawn outwards, as occurs during walking or sitting, if the pelvis is moved laterally, especially in lame females. The gas may also be secreted by the vagina. Usually the presence of the elastic fluid is only known by the noise consequent on its escape; but occasionally there is some uneasiness and pain, with difficulty in passing the feces and urine, and on passing the finger up the rectum the parietes are found in contact from the pressure of the dilated vagina. M. Lisfranc quite agrees with Frank that this affection is very common, though seldom complained of, because it very seldom produces sufficient inconvenience to require treatment; but the author has repeatedly observed air incarcerated in the vagina while examining patients labouring under other diseases of the uterine organs. When air is secreted by the vagina M. Lisfranc thinks the part is irritated or inflamed, and recommends an antiphlogistic plan of treatment. As the escape of air is not under the patient's control, she may be precluded from society; but M. Lisfranc has always succeeded in relieving this inconvenience by directing the patient to give exit to the gas by passing the finger into the vagina previously to mixing in company. (pp. 331-6.)

*Hydrometra or dropsy of the uterus.* Although a considerable number of cases of uterine dropsy have been recorded by very eminent practitioners, MM. Naegele and Stoltz (to whose opinions M. Lisfranc does not advert) deny the possibility of the occurrence of the disease except in connexion with pregnancy:—because the uterus is lined by a mucous and not a serous membrane; because its dense structure would present an insurmountable obstacle to its dilatation by the fluid; because the fluid would escape; and because, as they allege, no authentic case of the malady has yet been recorded. We shall neither discuss these theoretical objections, nor attempt to refute the latter assertion by a reference to any of the previously-reported cases; but merely observe that M. Lisfranc details two cases of hydrometra, in both of which "the absence of pregnancy was satisfactorily ascertained." (p. 347-8.) We need scarcely further advert to the contents of the chapter on this subject; but may observe that the author dwells on the necessity of removing congestion or chronic inflammation of the womb, if present, before any attempt is made to expel the fluid, whether by baths, purgatives, ergot of rye, or operation; for if this precaution be neglected, metro-peritonitis may be excited and prove fatal, as occurred in one case mentioned by M. Lisfranc, (p. 344,) from the untimely administration of ergot of rye. If, however, the symptoms are menacing and rapidly progressive, the womb must be emptied, notwithstanding the danger thereby incurred. The fluid is best evacuated by means of a gum elastic catheter; if this fails to overcome the obstruction, a silver canula or the finger must be employed, and occasionally we are driven to pass a canula through the cervix uteri. (p. 348.)

*Hydatids of the uterus and of the vagina.* Our observations on this chapter may be very brief. The great majority of writers maintain that hydatids of the uterus must be the result of conception; M. Lisfranc admits that the clustered hydatid, somewhat resembling a bunch of grapes, has always this origin, but that true hydatids may exist in the virgin uterus, and refers to a case recorded by Percy as indisputably proving this fact. (p. 355.) We may take this opportunity of noticing a statement frequently repeated in this volume: the author deprecates the use of intra-uterine injections in any case, as being eminently calculated to excite inflammation; and contrasts their influence in this respect with the fact that the introduction of instruments into the cavity of the womb is very rarely productive of bad consequences. (p. 361.)

*Inversion of the uterus.* M. Lisfranc agrees with those authors who consider distension and, perhaps, softening of the parietes of the uterus (whether from pregnancy, the presence of a polypus, &c.) an essential pre-requisite for the occurrence of inversion. Baudelocque, it is true, observed inversion of the uterus in a girl aged fifteen, who had certainly never been pregnant; but M. Lisfranc asks, may not the patient have been affected with retention of the catamenia, hydrometra, hydatids of the uterus, &c.? As to A. Dubois' suggestion, that Baudelocque, in this instance, mistook a polypus for inversion of the uterus, M. Lisfranc rejects it, as purely gratuitous and inconsistent with the great skill and judgment of that eminent practitioner. (p. 379.) The cases in which inversion of the uterus has been supposed to occur several days, weeks, or months after delivery, in M. Lisfranc's opinion, prove nothing, as the affection, he is satisfied, existed in the first instance, but was overlooked in consequence of being incomplete and causing little inconvenience; this position, we may observe, was long since advanced by Mr. Newnham, and perhaps by others, but is here given as if it originated with the author. (p. 383.) Though inversion of the uterus is too frequently fatal, from its immediate or remote consequences, or else produces great misery and distress, yet several cases have been recorded in which it produced but trifling inconvenience; and M. Lisfranc adds a very remarkable instance of this kind to those already recorded. A woman aged seventy, while convalescent from bronchitis, was permitted to remain in La Pitiè until she could be transferred to Salpétrière; "she exercised in the court of the hospital the greater part of the day; her appetite and digestion were excellent; she experienced no pain in the pelvis, and had no vaginal discharge." A second attack of bronchitis came on and terminated fatally. On dissection, the uterus was examined, as it were, accidentally, and M. Lisfranc "was utterly astonished on discovering complete inversion of the womb." (p. 379.) When the uterus becomes inverted after parturition, and the placenta is adherent to the organ, Clark, Burns, Blundell, Gooch, Newnham, &c. recommend that we should try and reduce the inversion without removing the placenta, chiefly from the apprehension of thereby augmenting the hemorrhage; Baudelocque, Gardieu, Capuron, Boivin and Dugès advise, on the contrary, the removal of the placenta; and M. Lisfranc advocates this practice, because the diminution of the volume of the tumour and the consequent facilitation of its reduction far counterbalances the danger of hemorrhage. (p 393.)

*Prolapsus of the uterus.* Displacements of the uterus, M. Lisfranc observes, are of extraordinarily frequent occurrence; and he entirely dissents from the generally-received opinion that they are *essential.* Prolapsus, anteversion, retroversion, and lateral deviations of the uterus are excessively rare, the author maintains, when the organ is exempt from hypertrophy; for among the immense number of females that the author has examined, he has met with but very few in whom these affections existed uncomplicated with augmentation of the size of the uterus. The direction of the displacement is governed by the situation of the hypertrophy. If the hypertrophy involves the entire circumference of the uterus, procidentia or prolapsus follows; if the front of the organ is affected there is anteversion, or if the posterior part of the womb is increased in size retroversion, on the contrary, occurs; does the induration occupy either side, the deviation is to that side. "The most elementary knowledge of mechanics enables us to see that a pyriform, slightly-flattened body, suspended in the pelvis from four cords, must, if it becomes sufficiently thickened, have its summit carried towards the symphysis pubis, and *vice versâ.*" It may be asked, is the hypertrophy coexisting with displacement of the uterus primary or secondary? but the fact that these displacements are rarely found essential or uncomplicated with hypertrophy, sufficiently answers this question; for as those displacements cause distress they would, on examination, be frequently detected in their first stage, before the hypertrophy had supervened, supposing it to be consecutive; but this is precisely what does not occur. In further proof of his opinion, the author appeals to the fact that if, when a diseased womb is hypertrophied, we treat and remove the hypertrophy the organ nearly resumes its natural position. (pp. 409-12.) But when the displacement is essential or primitive, hypertrophy of the womb may supervene as a secondary affection. (p. 417.) Essential or primary displacements of the uterus are generally very difficult to cure, because the constitution of the patient is greatly deteriorated, and because when the ligaments of the uterus are relaxed or stretched from any other cause than the sheer weight of the organ, it is difficult to restore them to their natural condition. If, however, hypertrophy of the uterus is the cause of the displacement, a cure can generally be effected. M. Lisfranc has not as yet met with a single case in which, when he could succeed in removing the hypertrophy, the womb did not resume very nearly its natural position, and occasionally the restoration was perfect. The treatment must therefore vary with the cause of the affection. (pp. 418-19.) We do not enter into the details respecting the treatment, because, as regards hypertrophy, they are more fully discussed in the chapter on *chronic metritis*, in the second volume of the work; and in other respects we find nothing novel except, perhaps, the following suggestion. Prolapsus of the uterus frequently impedes more or less the functions of the bladder and urethra, especially during pregnancy, and the emission of urine is greatly facilitated by passing the finger through the vagina, behind the symphysis pubis, the uterus is thus pushed back, and the compression it exerted diminished or even completely removed: the patient can herself readily practice this manœuvre. A frequent desire to pass urine commonly attends displacement of the uterus, pregnancy or simple hypertrophy of the organ; the patient may be compelled to pass water fifteen or twenty times in the night; M. Lisfranc has repeatedly relieved this symptom by directing a small enema, nearly cold and containing three or four grains of camphor, dissolved in yelk of egg, and a few drops of tincture of opium to be injected night and morning. (pp. 428-9.)

*Retroversion of the uterus.* This disease M. Lisfranc says was known to the ancients. But he adds, repeating the assertion of Sabatier and Boyer, it was first clearly described by Gregoire. We believe, on the contrary, that William Hunter, who is not mentioned by M. Lisfranc, was the first who published a case of the malady with a precise detail of the symptoms. (p. 431.) Boyer and others say that retroversion seldom occurs except during the early period of pregnancy; but this, according to M. Lisfranc, is a great error, the most frequent cause of the displacement being hypertrophy of the posterior part of the uterus. (p. 432.) The author has frequently met with cases simulating retroversion, but yet very distinct from it. In those cases the fundus of the uterus was perhaps inclined slightly backwards; the posterior surface of the organ was considerably hypertrophied; and the cervix being considerably congested, as in the third or fourth month of pregnancy, was elevated and applied against the symphisis pubis, but without losing its parallelism to the axis of the pelvis, while the rest of the womb in contact with the sacrum, and raised in consequence of the hypertrophy, lay in the same direction; consequently the fact of the cervix uteri being thrown very considerably forwards is not a proof of the existence of retroversion. (pp. 433-4.) Tumours behind the womb may elevate its cervix and depress the functions, and though the author has always found the womb in such cases slightly hypertrophied, yet we can always determine the nature of the case by ascertaining the condition of the cervix uteri. In fact the cervix uteri is never obliterated by a tumour external to the womb, as it is in pregnancy and in considerable hypertrophy of the organ, it is found in these cases in the same condition as in the third or fourth month of pregnancy; if then we have this condition of the cervix, coupled with a large tumour and absence of the signs of pregnancy, the tumour is external to the uterus. (pp. 435-6.) When retroversion is caused by hypertrophy, it is difficult and dangerous to restore the uterus to its natural position; but if we remedy the hypertrophy the retroversion often disappears. (p. 438.) In some cases of difficult reduction, Evrat succeeded by passing a slender cylinder of wood into the rectum, and pressing the fundus uteri upwards, while the cervix was depressed with two fingers in the vagina; M. Lisfranc has effected the reduction by passing a cylinder, of less diameter than that used by Evrat, up the vagina, and therewith elevating the body of the womb, its neck being at the same time pushed downwards and backwards with one finger. (pp. 441-2.) If adhesions exist between the neck of the uterus and the vagina, the displacement is generally supposed to be irremediable; but M. Lisfranc has divided those adhesions in several instances. In one case there was a slight interval between the cervix uteri and the vagina, and the cicatrix was divided with a blunt scissors directed on the index-finger; in two other cases the neck of the womb and the vagina were in close contact; the cervix uteri was seized with Museax's hook, and depressed until it projected externally to a sufficient extent, (a condition indispensable to the performance of the operation): in order to avoid perforating the rectum, bladder, or peritoneum, the dissection was performed very slowly and cautiously, and especial care was taken to carry the incision exclusively through the tissue of the uterus, but as close as possible to the adhesion; when the uterus was set free, the small portion of the uterus left adhering to the vagina was removed with a scissors curved on the flat. (pp. 433-4.) When retroversion of the uterus is complicated with pregnancy, and reduction is impossible, it may be necessary to puncture the uterus notwithstanding the danger of such a proceeding; M. Lisfranc prefers, with Viricel, performing this operation through the anterior wall of the rectum, as we thus penetrate the uterus at its most depending point; if, however, the puncture is practised through the vagina, we should perforate the body of the womb, and not the cervix, as this latter part is too thick and hard. (pp. 445-50.)

*Anteversion of the uterus.* This is universally stated to be greatly rarer than retroversion; but M. Lisfranc, on the contrary, maintains "from hundreds, he might say thousands of observations," that it is *infinitely* more frequent, a circumstance which is, he thinks, easily explained. In common with Boyer, Dugès, and others, (to whom he does not refer,) the author attributes the affection chiefly to the weight of the anterior parietes of the uterus being increased from hypertrophy. But the anterior surface of the uterus is more exposed than the posterior surface to injury, and therefore it is much more frequently hypertrophied; moreover, females pass urine very frequently, and thence the bladder being often empty the summit of the uterus readily falls forward, the displacement is further facilitated by the great prevalence of constipation in females, and the consequent accumulation of feces in the rectum. (p. 453.) As to the symptoms enumerated by M. Lisfranc, we shall only advert to one not generally sufficiently dwelt on: "The patients very frequently experience considerable uneasiness in the rectum; on examination it is ascertained that the rectum is perfectly healthy, but nevertheless it is often by no means easy to convince the patient that such is the case; they usually persist in saying

4295

that the womb is not affected, *that the disease is situated in the anus.*" (pp. 455-6.) We mention this the rather that more than one case has fallen within our own knowledge, in which certain of the tribe of rectum-doctors, far from trying to disabuse the patient of her erroneous idea, have, we must hope through ignorance, confirmed her in it. Complete anteversion is extremely rare; M. Lisfranc during a post-mortem examination met with one case, in which the capacity of the urinary bladder was diminished to a twentieth part of its natural size, and the fundus of the uterus was rotated forwards and downwards to such an extent that it had become nearly parallel to the axis of the pelvis. (p. 457.) The displacement is generally easily reduced, but occasionally great difficulty is experienced in accomplishing it. In one case where the operation seemed impossible, M. Lisfranc effected it by means of the cylinder of wood mentioned in the last paragraph. (p. 459.) A bandage or belt applied over a pad placed on the hypogastrium has a powerful influence in diminishing the pain caused by anteversion, and in facilitating its reduction even when the uterus does not rise above the symphysis pubis. After reduction a pessary, if it can be borne, may be useful. (p. 460.)

*Inclination or obliquity of the uterus.* This may occur when the womb is empty, or when it is distended either from pregnancy or a tumour. The presence of the sigmoid flexure of the colon and of the upper portion of the rectum at the left side and the prevalent habit of sleeping on the right side, render right lateral version the more common; the most frequent causes of the displacement when unconnected with pregnancy are, lateral hypertrophy, adhesion of the cervix uteri to one side of the vagina, and partial atrophy of the uterus occurring about the critical period of life. Besides causing leucorrhea, &c. this affection may prevent impregnation. The prognosis is favorable, except occasionally when acute inflammation exists. Reduction is easily effected, unless it is obstructed by the existence of adhesions or of hypertrophy, and a pessary frequently retains the uterus in situ. The indications of treatment may be collected from what has been said respecting other displacements of the uterus. (pp. 461-70.)

*Anteflexion and retroflexion, or incurvations of the uterus.* Incurvation of the uterus occurs rarely; M. Lisfranc has met with but six cases of it. We do not find anything added to what has been already stated respecting these affections by Denman, Deneux, Boivin and Dugès, and L'Heritier.

*On the mechanical means of treating displacements and enlargements of the uterus. Sponges.* Astringent injections are frequently employed concurrently with the various mechanical means destined to remedy displacements or hypertrophy of the uterus: M. Lisfranc has almost uniformly found such injections hurtful, when the patient was not much debilitated even though the parts were not inflamed; but they are, on the contrary, useful in relapsed languid constitutions, notwithstanding the existence of a slight amount of local inflammation. As a general rule the author prefers emollient injections. The introduction of sponge usually causes a good deal of uneasiness at first, though the genital organs may be quite free from inflammation, but nevertheless it should not be too hastily removed as the parts generally became accustomed to its presence. Neuralgic pains are very often removed by the employment of sponge, which should therefore be empirically applied when the existence of neuralgia is suspected. Sponges are useless in prolapsus uteri; they occasionally succeed in anteversion and retroversion; but are especially suitable in slight procidentia of the womb, and in prolapsus of the vagina, in which affections they often answer when pessaries fail. (pp. 475-82.)

*Pessaries* are employed to support a hypertrophied uterus, and to aid by the slight pressure they exert in dispersing the hypertrophy, (p. 475;) they are also fitted to counteract various displacements and relaxations of the uterus and of the vagina. Caoutchouc pessaries are greatly preferable to those manufactured from any other material except when we want to exert pressure on an hypertrophied uterus, in which case an ivory instrument is the best. (p. 499.) Pessaries are indiscriminately rejected by some practitioners, and as indiscriminately applied by others. The following are the rules, according to M. Lisfranc, which should govern their employment. They are contraindicated: 1st. When the patient expresses great repugnance to their use, (unless the circumstances of the case forbid delay,) because mental uneasiness exerts a most prejudicial influence on diseases of the uterus. 2d. When the genital organs are unusually sensitive or the seat of much pain, except in certain cases of neuralgia which are cured by the application of a pessary. 3d. When the instrument excites much pain, which sometimes does not happen for some time after its application, and is then almost uniformly due to the aggravation of a previously existing, but latent inflammation. 4th. When chronic vaginitis or metritis are present, and also for at least three weeks after they have been completely dispelled. 5. When the vagina or cervix uteri are ulcerated. Leucorrhea does not contraindicate the use of a pessary, except when it is complicated with chronic inflammation. (pp. 486-90.) Some practitioners recommend that the patient should be made to cough, sit, or even walk, in order to ascertain whether a pessary has been efficiently applied, but this is very injudicious, as being likely to aggravate the pain so commonly experienced during the first few days the instrument is worn; the patient should, on the contrary, be restricted to the recumbent posture for at least five or six days. When a large pessary is requisite, it very frequently cannot be tolerated if applied at once, but by first introducing a small instrument, and at suitable intervals replacing it by others of larger size, one of sufficient magnitude may ultimately be borne. (pp. 494-5.) Nothing can be more mischievous than to leave a pessary in the vagina for any considerable period; it should be removed every fifteen or twenty days, and after being thoroughly cleaned, immediately replaced. (p. 498.) There is a separate chapter *On the bad consequences resulting from the presence of pessaries in the vagina*, when they have been allowed to remain too long in that canal. On this chapter we need only observe that M. Lisfranc disapproves of removing a pessary during the existence of any considerable inflammation that its presence may have excited, as the manipulations necessary for its extraction, always aggravate the mischief: the inflammation should be reduced before the foreign body is extracted. (p. 510.)

We pass by the short and unimportant chapter on *Elevation of the uterus.*

*Preternatural fixity of the uterus.* An examination per vaginam will not always enable us to detect this affection; M. Lisfranc has not unfrequently met with patients in whom the uterus apparently retained its natural mobility, but on attempting to depress that organ for the purposes of operation, it was found to be quite immoveable; on the other hand, in cases where the womb when examined by the finger, seemed immoveably fixed, the cervix uteri was readily depressed so as to protrude through the vulva. (pp. 516-17.)

*Preternatural redness, excrescences (boutons), aphthæ, granulations of the cervix uteri.* Preternatural redness must not be confounded with the increased redness of the cervix uteri that exists during pregnancy, and for some days before and after menstruation. It occurs either in discrete or confluent patches, which are usually slightly prominent, or it may assume a punctated aspect resembling leech-bites; it may be superficial, or extend to some depth, and when limited to the surface, disappears momentarily on pressure; it more frequently occupies the posterior lip of the cervix, and usually occurs in adults before the critical period of life, after which it is rare. This affection is usually caused by congestion of the uterus, or by the contact of irritating liquids, whether secreted or introduced ab externo; it is usually permanent, but is sometimes fugitive; it may or may not be complicated with hypertrophy or inflammation, and when it exists for a considerable period, it commonly terminates in ulceration. The cervix uteri may seem perfectly healthy to the touch, when affected with preternatural redness, or even when eroded or superficially ulcerated; whenever therefore leucorrhea, anomalies of menstruation, &c. exist, the speculum should be applied, the red patches are then easily recognized, and in order to ascertain whether their surface is abraded, the part should be firmly wiped with a dossil of lint; if it is not stained with blood, there is no excoriation. . . . . Excrescences (boutons) often coexist with preternatural redness, or with hypertrophy of the cervix uteri; if neglected they may ulcerate, suppurate, or coalesce and form a tumour: they almost uniformly yield to antiphlogistics, combined with and followed by narcotics. . . . . When morbid redness is not complicated with inflammation or congestion, or when these complications have been dispelled, it should be cauterized with protonitrate of mercury, otherwise it may terminate in ulceration. A few cauterizations almost always suffice. The caustic should be applied with a camel-hair brush, which after being immersed in the solution, should be shaken or even gently wiped, so that a very minute portion of the fluid shall adhere to it; the neck of the uterus having been wiped dry through the speculum, the brush is once only, and as lightly and rapidly as possible passed over about one fourth of the diseased surface, and the speculum then filled with nearly cold water. When the redness is disseminated in isolated patches, it is sufficient to cauterize the entire surface of a single patch, provided it occupies about a fifth of the cervix. The treatment of granulations is exactly similar. (pp. 519-32.)

*Benign ulcers of the uterus.* Though ulcers are infinitely more common on the external surface of the cervix they occasionally occupy its inner surface, and cannot be then seen through the speculum, unless we dilate the cervix by gently opening the blade of a forceps previously passed into it. (p. 534.) According to M. Lisfranc, the affection is equally frequent at every age between the first appearance and ultimate suppression of menstruation, and its occurrence is not influenced by any peculiarity of temperament or of constitution; leucorrhea probably predisposes to the disease; and in hypertrophy of the womb, the existence of ulceration is the rule, its absence the exception. Displacements of the womb, a conical cervix, and painful menstruation are frequent causes of ulceration, and M. Lisfranc is satisfied that it is an hereditary disease; to this last opinion he attaches the utmost importance, because the simple, if neglected, is exceedingly prone, he says, ultimately to become malignant, and then the patient labours under cancer which is regarded as the more formidable and hopeless, because it is supposed to be of hereditary origin. Cancer, M. Lisfranc insists, is never hereditary, but simple ulceration, on the contrary, very frequently is; if an ulcer, indeed, is neglected, it may and often does, become cancerous, but it is always the ulceration, never the cancerous degeneration, that is hereditary. (pp. 535-8.) We pass by the description of the local appearances of ulcers of the uterus, which are very variable indeed. Their progress may be rapid, but is usually slow; the author has seen them remain stationary for ten years. They have little tendency to cicatrize, for though they often go on healing very rapidly for some time, the process is after a time arrested. Cicatrization is greatly slower in winter than in summer. (p. 540.) Some superficial ulcers bleed with the greatest facility; simply exposing them to the air, though the speculum does not touch them, causes blood to exude from their surface; this symptom indicates congestion of the womb, and though it usually yields to venesection and cauterization with protonitrate of mercury, still its presence gives grounds for apprehending the formation of a varicose, or a fungous, or an erectile tumour, or a fungus hematodes, for the author uses those four phrases to designate the degeneration which he says may follow this particular condition of ulcer. (pp. 541-3.) Simple ulcers of the womb occasionally simulate cancer so closely, both in appearance and in all their symptoms that the diagnosis can only be established by the result of the treatment. In other cases again, their progress is insidious and almost latent; there may be slight leucorrhea unattended with pain, or a sense of weight, and some trifling pain in the pelvis, there is no emaciation, no loss of appetite, and if an examination with the finger and the speculum is neglected, incurable cancer often intervenes. M. Lisfranc has seen several cases, which, in common with several very eminent surgeons, he pronounced to be incurable cancer of the uterus, and yet they perfectly recovered without any treatment save attention to cleanliness and observance of hygienic rules; he is inclined to think that ulcers of suspicious character, when they occupy an organ very liable to cancer, are too frequently assumed to be cancer; the odour *sui generis* of cancer is the only characteristic sign of the disease, but it is not constantly present, and sometimes never exists throughout the entire course of the most genuine cancer. (pp. 543-9, 553-8, 620.) The existence, the complete absence, or the peculiar character of the pain is of no value as a diagnostic sign; in simple ulcers the pain is often lancinating (p. 545), while cancer may run to a very advanced stage without more than a trifling amount of suffering having been sustained. (p. 567.) Non-malignant ulcers of the neck of the uterus are almost always curable. Congestion, induration, or hypertrophy of the womb retard the cure. The treatment usually occupies from three to six months, but may require a much longer period. (p. 539.) The general principles of treatment recommended by M. Lisfranc are, to remove congestion or inflammation when present, by rest, laxative enemata, narcotics by the mouth, rectum, or endermically, cold cataplasms on the abdomen, emollient vaginal injections, regulated diet, warm baths, venesection, and such other antiphlogistic treatment, as the circumstances of the case may require. Cold, whether applied locally or by general baths, and warm hip-baths are uniformly prejudicial, inasmuch as the former favour internal congestion, and the latter promote an afflux of blood to the pelvis. Immersing the upper extremities in hot water exerts, on the contrary, a very salutary derivative effect from the diseased organ. (pp. 557-68-71-4.) When congestion and inflammation have been dispelled, or when they do not exist, tonic and astringent injections, such as infusion and decoction of cinchona, oak-bark, rhatany, &c. or solutions of lead, zinc, or alum, may be thrown up the vagina. Pessaries are almost uniformly prejudicial when ulceration exists. Iodide of potassium administered internally is generally very advantageous when inflammation is either absent or very moderate, and is especially useful when the patient is debilitated. Bitters are often beneficial, as is iron also, when the blood is impoverished, but iron should not be continued too long, for though it invigorates the system, and restores the function of the stomach in particular, yet its protracted use almost uniformly causes dangerous congestion of the womb. (pp. 570-7.) But though these means may succeed, ulcers of the uterus, whether recent or old, seldom cicatrize, unless they are cauterized; caustic should not be applied when inflammation or congestion exists, except in those rare cases where the ulcer continues to extend, notwithstanding the employment of antiphlogistics and narcotics, or when an ulcer of bad character remains three or four weeks without any appearance of amendment. As a general rule, the caustic (protonitrate of mercury) should be applied in the manner described in the last section; but if the ulcer is deep, is covered with very luxuriant granulations, or has a very hard base the caustic should be applied more freely, but in no case should dossils of lint impregnated with the caustic solution, be left in contact with the ulcer for several minutes, as some recommend, for serious and too often fatal consequences may result from such practice; caustic should not be used for five or six days previous and subsequent to menstruation. It should usually be repeated every eighth day, but if the sore is indolent, its base hard, its surface granulating luxuriantly, the operation may be repeated twice or even thrice weekly. As cicatrization advances, the intervals may be prolonged, but if the ulcer begins to spread or remains stationary, the treatment must be resumed. However extraordinary it may appear, cauterizing one ulcer when several exist usually exerts a favorable influence on all. (pp. 581-97.) When copious leucorrhea or hemorrhage are running down the patient, or when the ulcer is spreading rapidly, particularly when it threatens to extend to the vagina, where it can scarcely ever be safely attacked either with caustic or the knife, and when the caustic proves inefficient, amputation of the neck of the uterus is indispensable. This practice was recommended by Dupuytren, and has on several occasions been successfully adopted by M. Lisfranc. (p. 557.)

*Cancer of the uterus.* Cancer of the uterus very seldom exists without ulceration. According to Bayle, it almost invariably commences as a simple ulcer, and M. Lisfranc entirely coincides in this opinion.

Occult cancer, which is fortunately a very rare disease, occurs indifferently on the cervix or on the body of the uterus, and it is very difficult or rather impossible to distinguish it from other indurated tumours of that organ, for lancinating pains, great hardness, a mammillated surface, &c. and the same train of general symptoms may equally attend both; and further, not only do simple indurations tolerably often present all the phenomena of *occult cancer*, but this latter affection occasionally appears under the most benign aspect: the diagnosis is therefore impossible, and from this circumstance, coupled with the fact that occult cancer of the uterus is an extremely rare disease, the treatment should always be grounded on the assumption that the tumour may be curable. If fluctuation is detected in the neck of the uterus, the tumour should always be allowed to open spontaneously, unless the violence of the symptoms forces us to evacuate the fluid by an incision; for if we unfortunately cut into an occult cancer, from that moment the patient becomes a victim to all the miseries of open cancer, which also uniformly runs an extremely rapid course. (pp. 597-609.) As already stated in the last paragraph, simple ulcers of the uterus may be attended with every symptom of open cancer, except the odour *sui generis* of the latter disease. (pp. 603, 621.) M. Lisfranc's opinion as to the age at which cancer of the uterus most frequently occurs is, we believe, peculiar to himself. He believes that it is most liable to affect females between the ages of eighteen and thirty-five, and that the general opinion that it most commonly sets in about the period of the cessation of menstruation is decidedly erroneous; he avers that he saw a girl aged fifteen affected with cancer of the cervix uteri. (p. 610.) This statement seems only to relate to cancer supervening on a simple ulcer, for we are elsewhere informed that the author has never observed *occult* cancer, except in females past forty. (p. 598.) M. Lisfranc, as we have seen repeatedly, insists that cancer of the uterus is very rarely a primary affection, and here again reverting to this doctrine, he inculcates as a rule of practice, "that the slightest symptoms either indicating uterine derangement, or even appearing to be connected therewith, imperatively require that an examination per vaginam should be made, both with the finger and the speculum." (p. 613.) It is owing to the neglect of this precept that incurable cancer has not unfrequently been detected, when its existence was antecedently not even suspected, and it is such cases that have given origin to the alarming doctrine that cancer sometimes occurs in a perfectly latent form: M. Lisfranc is however convinced that there is no disease of the uterus, be it what it may, which is not uniformly and in every case attended with symptoms perfectly appreciable and distinctly indicating that the uterus may be diseased; whenever this is even *suspected* to be the case, an examination should be forthwith instituted, and M. Lisfranc is decidedly of opinion that since this practice has been somewhat generally adopted in Paris, incurable diseases of the uterus have become greatly less frequent. Formerly eight or ten females, scarcely one of whom had been previously examined, applied every week at La Pitié labouring under cancer of the womb; at present not more than two or three are seen in a month. M. Lisfranc seems to anticipate a period when malignant diseases of the womb will scarcely be met with, in consequence of the curable affection in which he believes they almost uniformly originate, being detected and treated in their early stage. When the ulcer is not too large, it should be treated as if it was curable; protonitrate of mercury should be applied in the manner already described, but more freely and more frequently; and iodide of potassium should be administered internally. Even in apparently desperate cases, where the use of caustic is out of the question, iodide of potassium should be prescribed, as it sometimes unexpectedly effects a cure, and still more frequently allays pain and retards the progress of the disease. Caustic sometimes alleviates, but more frequently exasperates the pain, and should not therefore be applied, if the suffering is great, and uncontrollable by narcotics. (pp. 623-8.)

*Amputation of the neck of the uterus.* It is well known that M. Lisfranc, in June 1834, presented a memoir to the Academy of Sciences, in which he recommended amputation of the cervix uteri in certain cases of cancer of that part, and gave a very flattering account of the success of the operation, alleging for example, that 84 out of 99 patients recovered, that hemorrhage seldom occurred, and when it did, was always readily restrained, &c. It is equally well known that M. Pauly (Maladies de l'Uterus, 8vo, Paris, 1836,) accused M. Lisfranc of deliberate falsehood, of suppression of truth, stating that 53 operations only had been performed, that several unsuccessful cases which had been treated in hospital were not recorded, that of 19 private patients, whom M. Pauly names, *one* only recovered, and *four* died within twenty-four hours, that in 12 patients the disease returned immediately, that of 9 patients operated on in his own presence, 6 had alarming hemorrhage, and 3 died in less than twenty-four hours; moreover, M. Pauly specified by name several individuals on whom M. Lisfranc said he had operated, though, in point of fact, no operation had been performed on any of them, and added much more to the same effect, which we need not recapitulate. Now M. Lisfranc never made any reply to M. Pauly's allegations, and we can scarcely say that he does so in the present work. We are indeed told that he never *published* his memoir; that it was a *secret* document intended to support his claims, when a candidate for election at the Institute; that it was obtained from the archives of that body, without his knowledge, and was shamefully misrepresented; that he has since *withdrawn* the memoir, but will not enter into explanations of the misrepresentations he complains of, because "professional honour imperatively forbids such a course. I would not for any consideration betray the secrets of my patients." (pp. 666-7.) M. Lisfranc, it would appear, thinks least said soonest mended, and it would have been better for his character to have fully acted up to that maxim, and not have alluded at all to this ugly business. His own version of the transaction comes simply to this. He knew that his memoir could never bear publication, but he hoped that it might, as a *secret* document, influence some members of the academy to vote for his election. After such a humiliating exposure of misrepresentation and fraud, we can of course attach but little importance to statements respecting this operation, but as rash operative interference is one of the besetting sins of the present day, it will be useful to show how greatly M. Lisfranc has modified his practice respecting this operation, though he would fain make it appear that his opinions have undergone no change.

M. Lisfranc says that amputation of the neck of the uterus may be attempted: 1. When well characterized cancer extends too deeply to be destroyed by caustic. 2. When the disease has not spread above the attachment of the vagina to the uterus. 3. When, though the existence of carcinoma is doubtful, the health is rapidly giving way in spite of treatment. (p. 636.) 4. Hypertrophy of the body of the uterus does not contraindicate the operation, unless the tumour is very hard, or about double the natural size of the organ. 5. Even if the tumour exceeds this size, yet if it is scarcely painful, the cancer of the cervix may be removed, as the operation affords a chance of recovery, seeing that the body of the uterus is often only affected with simple white induration. (pp. 637-8.) The fact stated by Bayle that the neighbouring lymphatic glands and adjacent parts are much less likely to become affected in cancer of the uterus, than in cancer of any other organ, is a circumstance very favorable to the success of the operation. (pp. 609, 638.) 7. As is also the fact that cancerous degeneration often affects but a limited portion of the tumour. (p. 638.) 8. Moderate enlargement of the ovaries does not forbid the operation. 9. Neither does the existence of enlarged lymphatic glands, provided they are recent, few, and not too bulky or hard. (p. 639.) When the disease has been but partially removed, the portion left behind has sometimes been cauterized, but this proceeding is usually fatal, and M. Lisfranc was thence induced in several cases to dissect out the morbid structure from the parietes of the womb, and he avers that he in this way succeeded in several cases. (pp. 631-42-49.) Hemorrhage, we are told, rarely follows the operation, and when it does is always easily restrained by plugging; the plug should not, however, be too hastily applied, as a moderate loss of blood tends to prevent metritis, unless the patient was very feeble, or the hemorrhage sudden, M. Lisfranc would not plug until twelve ounces of blood were lost. (pp. 651-2.) The wound is often cicatrized in six or eight weeks, but may not heal for several months. The operation in no way interferes with utero-gestation; parturition is attended with less difficulty than previously, and the patient menstruates as usual. (p. 659.) From the foregoing abstract of the most important statements made by M. Lisfranc respecting this operation, it might be inferred that it is rather frequently indicated, but this we are told is not the case; M. Lisfranc himself now performs it very rarely, not it appears from any distrust in its safety or efficacy, but simply because cancer of the uterus is much less frequent now than it was a few years since. We trust that we need not occupy space in exposing this miserable and uncandid subterfuge.

The sections on *extirpation of the uterus*, (pp. 667-75,) on *contraction and obliteration of the Fallopian tubes*, (pp. 675-7,) and on *inflammation of the ovaries, of the Fallopian tubes, and of the ligaments of the uterus*, (pp. 677-91,) do not contain anything requiring to be noticed here, and with respect to the chapter on *ovarian dropsy*, (pp. 691-720,) we find but one point necessary to advert to. M. Lisfranc disapproves of extirpating ovarian cysts. Unless the tumour is very moveable, we have no reason to assume that extensive adhesions do not exist, but this character of mobility cannot be satisfactorily detected unless the tumour is very small; and if the tumour is very small, experience tells us that it may remain stationary, that its progress may be very slow, and that even should it increase greatly, the patient may nevertheless sometimes live a long time without enduring much suffering; against these *chances* we have to balance some *extraordinary* rare cases of successful operation, which, in M. Lisfranc's opinion, do not turn the scale, an opinion in which we most fully coincide. (p. 711.)

*On some tumours adjacent to, but not connected with the genital organs.* Cancer high up the rectum may push the uterus forwards, and if we confined ourselves to an examination per vaginam, we might suppose that the patient was merely affected with displacement and hypertrophy of the womb; but the true nature of the case is at once detected on passing the finger up the rectum. (p. 720.) Tumours, whether the result of inflammation or of any other cause, occasionally form external to the womb; they often acquire a considerable size, cause extreme pain, and are accompanied with almost all the symptoms of disease of the uterus. In most of those cases, the diagnosis is extremely difficult, not to say impossible, for the tumour is confounded with and seems to be a part of the uterus; but M. Lisfranc has observed that when the womb is *considerably* enlarged, the cervix is almost uniformly shortened or even effaced; whenever then the neck of the uterus retains its natural length, there is reason to believe that the tumour is external to the womb. (pp. 721-2.) Diseases of the womb are frequently attended with exquisitely acute pain, which cannot be allayed by the most powerful narcotics or other remedies; in such cases magnetism is a potent means of relief: "far be it from me" says M. Lisfranc, "to admit the reveries of the magnetisers; but beyond all doubt the influence of mesmerism on the nervous system of females thus circumstanced may be extremely salutary; I am satisfied of this from the result of numerous cases; I have seen the pains disappear as if by enchantment." (pp. 724-5.)

*Some considerations on hemorrhoids, and on their treatment.* M. Lisfranc has scarcely ever seen hemorrhoids consisting of erectile tissues, and he thinks they are very rarely formed by dilated veins. He does not give a very clear explanation of what he conceives to be their usual structure, but seems to regard them as hypertrophied cellular tissue, either congested or infiltrated with blood. (pp. 731-3.) When hemorrhoids produce very considerable distress either from being irreducible or descending and causing much pain every time the patient goes to stool, and the ordinary palliative treatment fails to give relief, M. Lisfranc recommends the patient to be bled at the arm, one or several times, according to his strength, state of health, &c., and then to direct a shower-douche of tepid water on the anus; this is to be replaced after a time by a similar douche of Barege water, and finally by a douche from a single jet, which, on occasion, may be passed up the rectum; "these means are heroic; I have seldom seen them fail, and we are thus enabled to avoid the performance of a dangerous operation." (p. 739.) When compelled to excise hemorrhoids, M. Lisfranc proceeds thus:—Having fixed the tumour with a hook, he circumscribes it with two semilunar incisions, and then, instead of cutting it away with one or two strokes of a scissors, he dissects it slowly off by several successive incisions; as the parts are kept down, any vessels that may be opened can be easily twisted or tied, and before completely detaching the tumour, we should ascertain whether an artery runs in the portion still adherent, by grasping it between the finger and thumb. The operation should be terminated very deliberately, and but a minute portion of the tissues be divided at once. The author says that by proceeding in this way, we incur no danger of hemorrhage. (pp. 743-4.)

Our readers are now in a condition to form a fair estimate of the merits of the present volume. We have been very sparing of comment, because we preferred curtailing our own observations to omitting anything that seemed important in a work purporting to contain the matured experience of a practitioner of great reputation, and who has enjoyed vast opportunities of observing diseases. That the present volume contains much that is valuable, we do not pretend to deny, if it did not we should not have occupied so much of our time and space with it; but it is disfigured with numerous and unpardonable faults. Nothing but a perusal of the original could convey an idea of the inflated and exaggerated laudation of self, and of the acrimonious depreciation of others that pervade the entire book. M. Lisfranc, to believe himself, is the great regenerator of modern practice; others are the blind slaves of an antiquated routine; he alone has laid the foundation of the true precepts of scientific surgery, by combining in harmonious union anatomy, physiology, and pathology, and being the first to conjoin medical and surgical therapeutics in the treatment of disease. The author also indulges in the most ludicrous over-estimate of the merits of the most trifling point that he conceives to originate with himself. We believe we have extracted almost every prominent statement in the volume, and the profession can judge how far the author overrates his own achievements.

---

